# Supplementary figures and images for: Histology-specific long-term oncologic outcomes in patients with epithelial ovarian cancer who underwent complete tumor resection: The implication of occult seeds after initial surgery
Source: PLoS One. 2024 Nov 25;19(11):e0311421. doi: 10.1371/journal.pone.0311421 (PMC11588254; doi:10.1371/journal.pone.0311421)

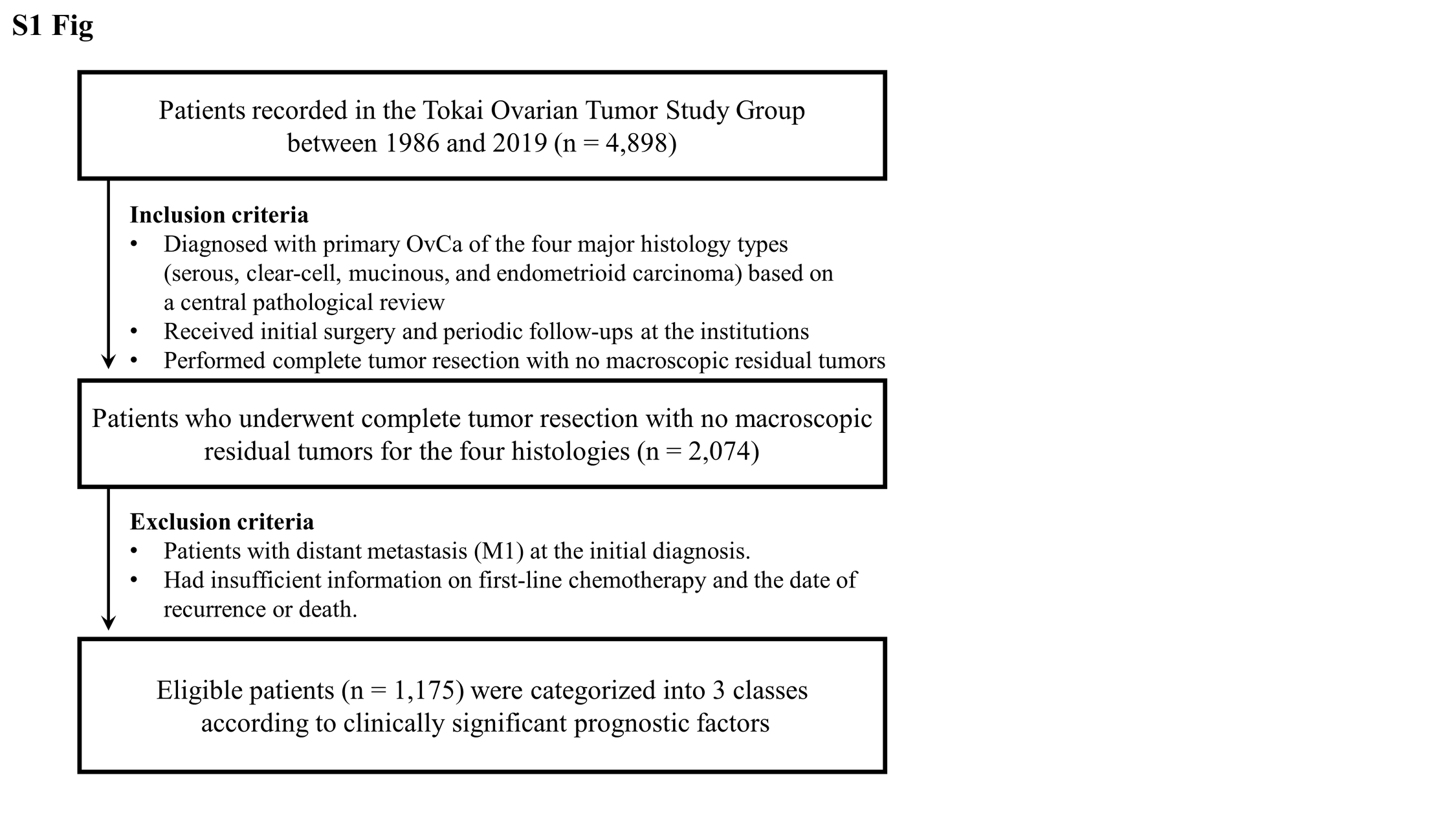

Supplement: S1 Fig — Flowchart of the selection of patients with OvCa who underwent complete tumor resection at the initial surgery from the database of the Tokai Ovarian Tumor Study Group. (TIF) [file pone.0311421.s001.tif]

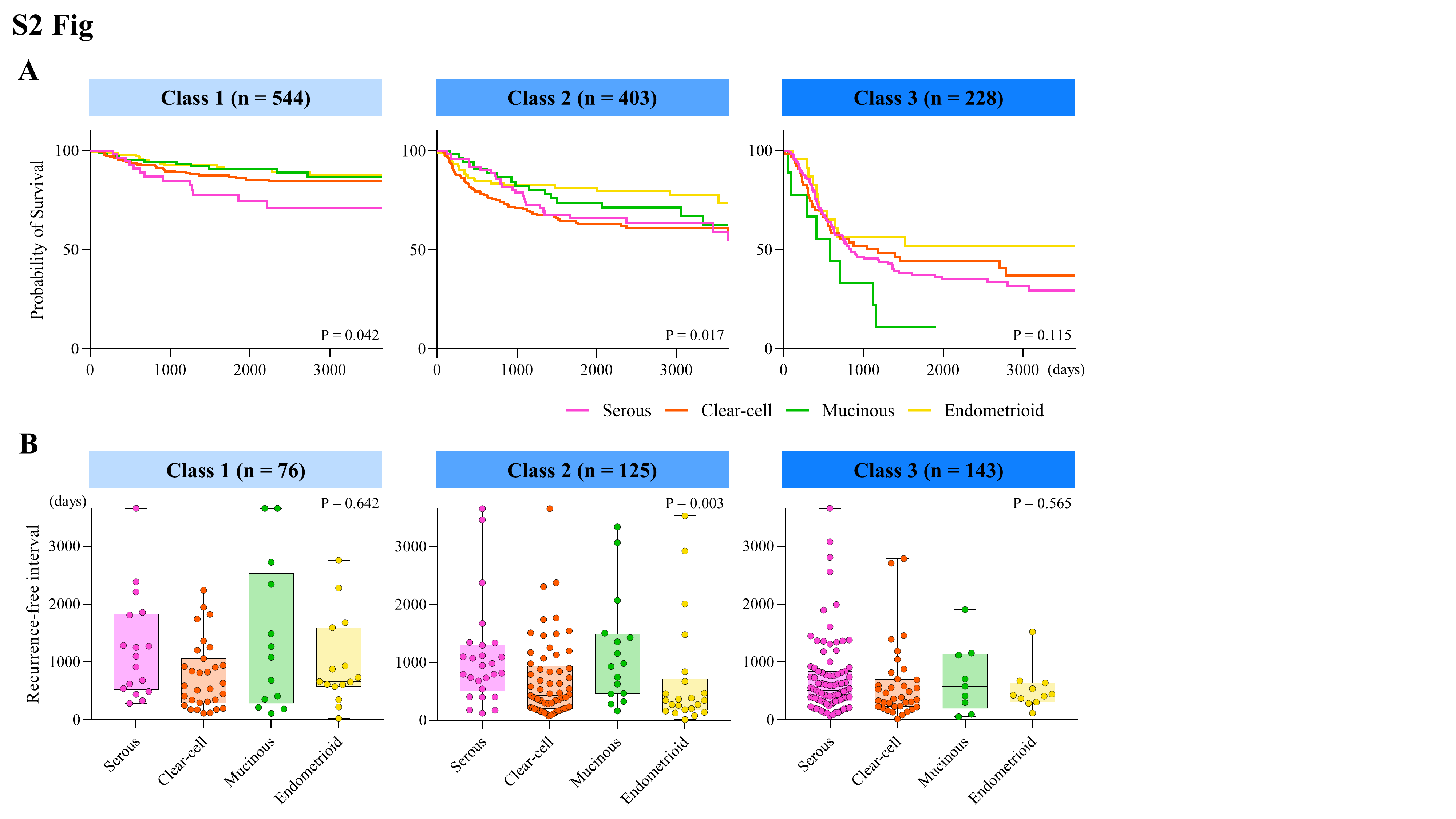

Supplement: S2 Fig — Kaplan-Meier curves of recurrence-free survival stratified by histology types and the 3 classes (A). P-values were estimated by the Log-rank test. Recurrence-free intervals for patients who developed recurrent tumors were compared among the histology types with the stratification of the 3 classes (B). P-values were estimated by the Kruskal-Wallis test. (TIF) [file pone.0311421.s002.tif]

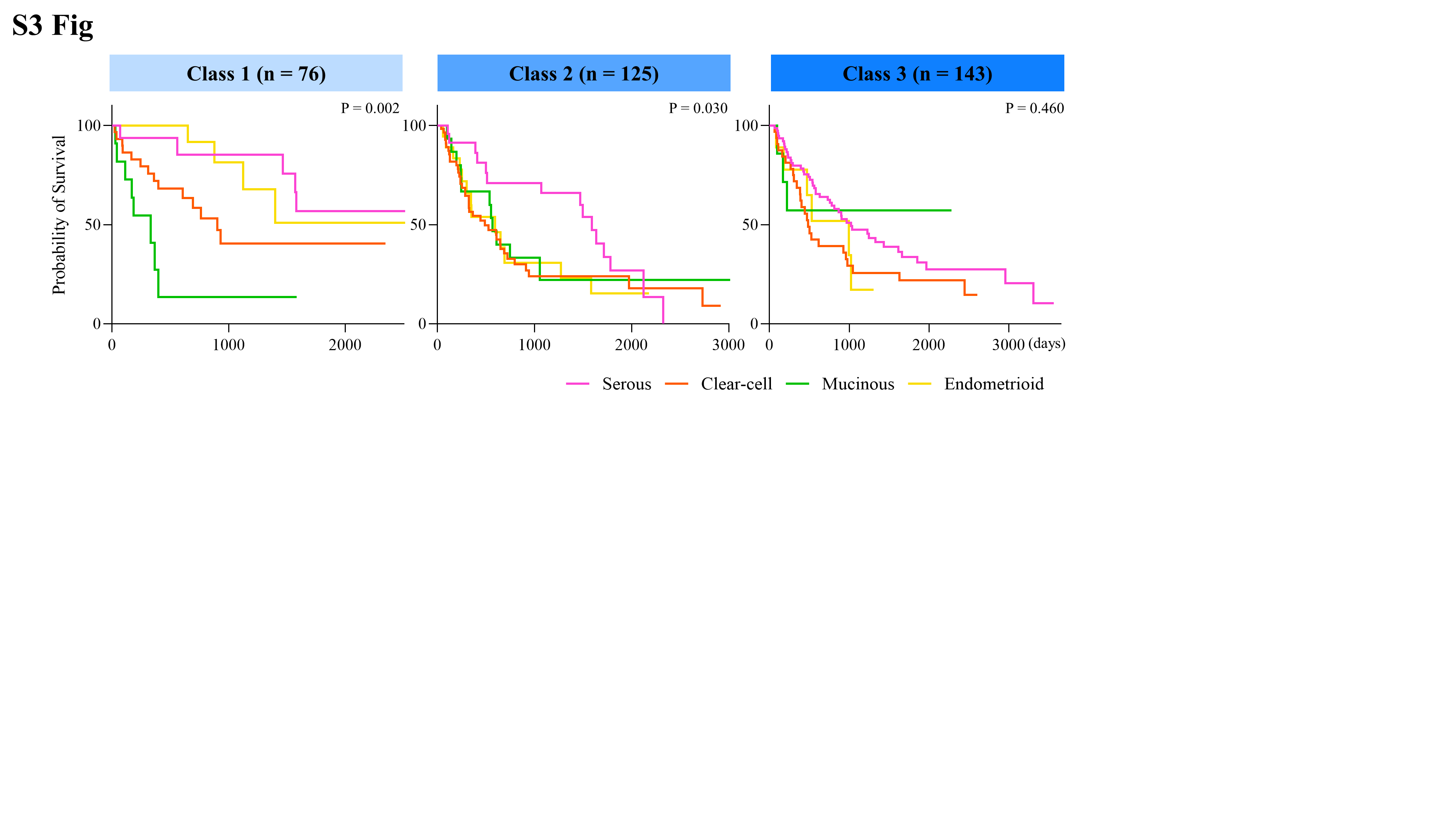

Supplement: S3 Fig — Kaplan-Meier curves of post-recurrence survival stratified by histology types and the 3 classes. (TIF) [file pone.0311421.s003.tif]
