# Supplementary material for: Histology-specific long-term oncologic outcomes in patients with epithelial ovarian cancer who underwent complete tumor resection: The implication of occult seeds after initial surgery
Source: PLoS One. 2024 Nov 25;19(11):e0311421. doi: 10.1371/journal.pone.0311421 (PMC11588254; doi:10.1371/journal.pone.0311421)
Supplement: S1 Table — Abbreviations: HR, hazard ratio; CA, cancer antigen. * Logarithmically transformed when analyzed. (DOCX) [file pone.0311421.s004.docx]

**S1 Table. Cox regression analysis for assessing factors associated with recurrence-free survival (n = 1,175).**

|  | **Univariate** | | **Multivariate** | |
| --- | --- | --- | --- | --- |
| **Categories** | **HR (95%CI)** | **P value** | **HR (95%CI)** | **P value** |
| Histology |  |  |  |  |
| Serous | reference |  | reference |  |
| Clear-cell | 2.028 (0.848–4.852) | 0.112 | 1.125 (0.843–1.500) | 0.424 |
| Mucinous | 1.041 (0.184–5.898) | 0.963 | 0.837 (0.535–1.310) | 0.436 |
| Endometrioid | 2.134 (0.714–6.375) | 0.175 | 0.618 (0.425–0.899) | 0.012 |
| Age |  |  |  |  |
| < 50 years | reference |  | reference |  |
| ≥ 50 years | 0.686 (0.321–1.469) | 0.332 | 1.201 (0.940–1.535) | 0.143 |
| Stage |  |  |  |  |
| I | reference |  | reference |  |
| II | 0.444 (0.132–1.494) | 0.190 | 1.403 (0.970–2.030) | 0.072 |
| III | 0.610 (0.262–1.424) | 0.253 | 3.330 (2.422–4.281) | <0.001 |
| CA-125* | 1.254 (1.008–1.561) | 0.042 | 1.063 (0.988–1.143) | 0.101 |
| Surgery |  |  |  |  |
| Uterine-preserving surgery | 0.281 (0.069–1.145) | 0.077 | 1.223 (0.827–1.814) | 0.311 |
| Full-staging lymphadenectomy | 0.597 (0.294–1.212) | 0.154 | 0.835 (0.660–1.057) | 0.133 |
| Positive ascites cytology | 1.179 (0.537–2.423) | 0.655 | 1.580 (1.245–2.005) | <0.001 |
| Ascites volume |  |  |  |  |
| < 100 mL | reference |  | reference |  |
| ≥ 100 mL | 0.579 (0.275–1.221) | 0.151 | 1.386 (1.090–1.762) | 0.008 |
| Chemotherapy | 0.434 (0.120–1.570) | 0.203 | 1.138 (0.742–1.745) | 0.554 |

Abbreviations: HR, hazard ratio; CA, cancer antigen.

* Logarithmically transformed when analyzed.
