# Supplementary material for: Histology-specific long-term oncologic outcomes in patients with epithelial ovarian cancer who underwent complete tumor resection: The implication of occult seeds after initial surgery
Source: PLoS One. 2024 Nov 25;19(11):e0311421. doi: 10.1371/journal.pone.0311421 (PMC11588254; doi:10.1371/journal.pone.0311421)
Supplement: Attachment — Submitted filename: reviewer.doc [file pone.0311421.s005.doc]

Histology-specific long-term oncologic outcomes in patients with epithelial ovarian

cancer who underwent complete tumor resection: The implication of occult seeds after

initial surgery

The study investigated the histology-specific long-term oncologic

outcomes of patients with OvCa undergoing complete tumor resection using a large

scale patient cohort accumulated in multiple institutions under a central pathological

review system. There are two questions

1. The title is histology-specific long-term oncologic outcome of patients with OvCa, but the analyzed factors included stage, ascits, and cytology. To our knowledge,the metastasis of lymph nodes is also included in the staging, and the prognosis is quite different between IIIA or IIIC. So to only difine stage III may not be exact.
2. The results have some predictive value for prognosis, but no clear for treatment.
