# Supplementary material for: Histology-specific long-term oncologic outcomes in patients with epithelial ovarian cancer who underwent complete tumor resection: The implication of occult seeds after initial surgery
Source: PLoS One. 2024 Nov 25;19(11):e0311421. doi: 10.1371/journal.pone.0311421 (PMC11588254; doi:10.1371/journal.pone.0311421)
Supplement: Attachment — Submitted filename: Response to Reviewers.docx [file pone.0311421.s006.docx]

Response to Reviewers

Reviewer #1: I read with great interest the Manuscript titled " Histology-specific long-term oncologic outcomes in patients with epithelial ovarian cancer who underwent complete tumor resection: The implication of occult seeds after initial surgery”, topic interesting enough to attract readers' attention.

Authors should clarify some point and improve the discussion citing relevant and novel key articles about the topic:

- I suggest a round of language revision, in order to correct few typos and improve readability.

- Considering topic and results if this study, it would be interesting to add few lines about

current evidence of the role of lymphadenectomy in advanced ovarian cancer, considering the advantages and limitations of this procedure. I would be glad if the authors discuss this important point, referring to PMID: 32036457

- In advanced ovarian cancers patients is crucial evaluate morbidity and mortality of cytoreduction considering risk and benefits of this surgical complex procedure. Considering results of this study, the authors should discuss solid evidence about hepatobiliary involvement and the high complex multivisceral surgery it requires. I would be glad if the authors discuss this important point, referring to PMID: 32779050

Considered all this points, I think it could be of interest for the readers and, in my opinion, it deserves the priority to be published after minor revisions

A: Thank you very much for your valuable feedback.

We have added the topics and references for lymphadenectomy and cytoreduction in advanced ovarian cancers patients to the discussion as you suggested. And we have corrected typos and omissions to improve readability. In addition, some colors in the graphs of Figures S2 and S3 have been corrected.

Reviewer #2: 1. The title is histology-specific long-term oncologic outcome of patients with OvCa, but the analyzed factors included stage, ascits, and cytology. To our knowledge,the metastasis of lymph nodes is also included in the staging, and the prognosis is quite different between IIIA or IIIC. So to only difine stage III may not be exact.

A: Thank you for pointing this out. As you pointed out, the prognosis may differ between stage IIIA and stage IIIC. We analyzed our data and found that the prognosis tended to be worse in stage IIIC, although the difference was not significant (p = 0.9345).

We reviewed the data in detail and found that there were 32 stage IIIA cases, of which 9 had only positive lymph nodes (IIIA1) and 23 had microscopic dissemination (IIIA2, including one lymph node-positive case).

IIIA1 (9 cases) were small in number and had a short follow-up period, but no deaths were observed. On the other hand, in IIIA2 (23 cases), 10 deaths (43.5%) were observed, similar to those in IIIB and IIIC.

On the other hand, significant differences are confirmed in the comparison between Stage I, II and IIIA (p < 0.001).

Therefore, although positive lymph node metastasis may affect prognosis, it is assumed that peritoneal dissemination may be a worse prognostic factor.

In this study, we categorized patients using ascites volume and ascites cytology as clinical factors that are potentially related to the dissemination progression.

Although it may be more accurate to add information on lymph node metastasis and dissemination, since this study was conducted to examine the prognostic and recurrence profile of each histology in patients who had complete resection, we think that the effect of stage IIIA1 on those analyses is small.

However, stage IIIA1 cases may have a unique course, and we would like to consider this issue in cases with only positive lymph nodes metastasis as a topic for future research. Thank you very much for your valuable comments. We have added the above information to Limitation.

2.The results have some predictive value for prognosis, but no clear for treatment.

A: Thank you for your precious comments. We think that this study is a retrospective analysis and is not suitable for prospective treatment prediction. From the real-world data in this study, we found ascites volume, ascites cytology, and stage may be useful in predicting prognosis. We also think that our data revealed the recurrence patterns and prognostic characteristics of post-recurrence treatment for each histology. We hope that these data will be helpful in clinical practice.
