# Supplementary material for: Histology-specific long-term oncologic outcomes in patients with epithelial ovarian cancer who underwent complete tumor resection: The implication of occult seeds after initial surgery
Source: PLoS One. 2024 Nov 25;19(11):e0311421. doi: 10.1371/journal.pone.0311421 (PMC11588254; doi:10.1371/journal.pone.0311421)
Supplement: Attachment — Submitted filename: Response to Reviewers.docx [file pone.0311421.s007.docx]

Response to Reviewers

Reviewer #1: The quality of the manuscript has improved thanks to the changes made. I think it could be of interest to the readers and, in my opinion, it deserves the priority to be published.

A: Thank you for your comment.

It has improved thanks to your appreciated suggestions.

Reviewer #2: The report has some predictive value for prognosis of epithelial ovarian cancer. Except for the lymphadnectomy, the author also raised up that chemotherapy has no relationship with the recurrence of the disease. I think we need more informations or subgroup analysis to explain the result.

A: Thank you for your valuable comments. In the multivariate analysis of this cohort, chemotherapy was not among the factors that significantly influenced recurrence-free survival. However, this does not mean that chemotherapy has no effect on recurrence. Because this cohort included stage I, II and III patients with no residual tumor, we identified three factors that we thought were common to these patients and used them to stratify the cohort. Therefore, it is difficult to analyze the relationship between chemotherapy, relapse, and histologic type in this cohort and subgroup.

However, the impact of chemotherapy on microscopic occult tumor metastasis is an important concern, and we would like to investigate the proposed issue in a further study.
