# Supplementary material for: Histology-specific long-term oncologic outcomes in patients with epithelial ovarian cancer who underwent complete tumor resection: The implication of occult seeds after initial surgery
Source: PLoS One. 2024 Nov 25;19(11):e0311421. doi: 10.1371/journal.pone.0311421 (PMC11588254; doi:10.1371/journal.pone.0311421)
Supplement: Attachment — Submitted filename: Response to Reviewers.docx [file pone.0311421.s008.docx]

Response to Reviewers

Journal Requirements:

Please review your reference list to ensure that it is complete and correct. If you have cited papers that have been retracted, please include the rationale for doing so in the manuscript text, or remove these references and replace them with relevant current references. Any changes to the reference list should be mentioned in the rebuttal letter that accompanies your revised manuscript. If you need to cite a retracted article, indicate the article’s retracted status in the References list and also include a citation and full reference for the retraction notice.

Additional Editor Comments:

While revising your submission, please upload your figure files to the Preflight Analysis and Conversion Engine (PACE) digital diagnostic tool, https://pacev2.apexcovantage.com/. PACE helps ensure that figures meet PLOS requirements. To use PACE, you must first register as a user. Registration is free. Then, login and navigate to the UPLOAD tab, where you will find detailed instructions on how to use the tool. If you encounter any issues or have any questions when using PACE, please email PLOS at <a href="mailto:figures@plos.org">figures@plos.org. Please note that Supporting Information files do not need this step.

In compliance with data protection regulations, you may request that we remove your personal registration details at any time. (Remove my information/details). Please contact the publication office if you have any questions.

Answer:
Thank you very much for your suggestion.

We have reviewed the reference list and verified that it is complete and correct.

We uploaded Figures 1-4 to PACE and no image problem was detected.

Thanks.
