# Supplementary material for: Histology-specific long-term oncologic outcomes in patients with epithelial ovarian cancer who underwent complete tumor resection: The implication of occult seeds after initial surgery
Source: PLoS One. 2024 Nov 25;19(11):e0311421. doi: 10.1371/journal.pone.0311421 (PMC11588254; doi:10.1371/journal.pone.0311421)
Supplement: Attachment — Submitted filename: Response to Reviewers.docx [file pone.0311421.s009.docx]

Response to Reviewers

Journal Requirements:

Please review your reference list to ensure that it is complete and correct. If you have cited papers that have been retracted, please include the rationale for doing so in the manuscript text, or remove these references and replace them with relevant current references. Any changes to the reference list should be mentioned in the rebuttal letter that accompanies your revised manuscript. If you need to cite a retracted article, indicate the article’s retracted status in the References list and also include a citation and full reference for the retraction notice.

Additional Editor Comments:

Please perform a final proof read of the document and correct typographic errors.

Examples where these currently occur include:

- Abstract

- Line 267

- Title of Reference List

Answer:
Thank you very much for your pointing.

We have corrected typographical errors in the manuscript.

We have also removed duplicate references and renumbered references in the reference list.

We uploaded Figures 1-4 to PACE and no image problem was detected.

Thanks.
